# Biological Pests Management for Sustainable Agriculture: Understanding the Influence of *Cladosporium*-Bioformulated Endophytic Fungi Application to Control *Myzus persicae* (Sulzer, 1776) in Potato (*Solanum tuberosum* L.)

**DOI:** 10.3390/plants11152055

**Published:** 2022-08-05

**Authors:** Oussama A. Bensaci, Khamsa Rouabah, Toufik Aliat, Nadia Lombarkia, Vadim G. Plushikov, Dmitry E. Kucher, Petr A. Dokukin, Sulukhan K. Temirbekova, Nazih Y. Rebouh

**Affiliations:** 1 Laboratory of Improvement of the Phytosanitary Protection Techniques in Mountainous Agrosystems (LATPPAM), Agronomy Department, Institute of Veterinary and Agricultural Sciences, Batna 1 University, Batna 05000, Algeria; 2 Higher National School of Forests, Khenchela 40000, Algeria; 3 Department of Environmental Management, Peoples’ Friendship University of Russia (RUDN University), 6 Miklukho-Maklaya Street, 117198 Moscow, Russia; 4 All-Russian Research Institute of Phytopathology, Bolshye Vyazyomy, Odintsovo District, 143050 Moscow, Russia

**Keywords:** biocontrol, conservation agriculture, invert emulsion, insecticidal activity, peach potato aphid

## Abstract

The potato is a staple food crop worldwide and the need for this product has increased due to the burgeoning population. However, potato production is highly constrained by biotic stress interference, such as *Myzus persicae* Sulzer, which causes serious yield losses and thus minimizing production income. The current study aims to investigate the effect of different formulations prepared as an invert emulsion with different concentrations of fungal culture filtrates derived from three endophytic fungi (genus *Cladosporium*) against *Myzus persicae*. All formulations have demonstrated an aphicidal activity, which increases with the increasing concentration of fungal filtrates. Furthermore, it has been noted that chitinolytic activity recorded for 12 days is important in *Cladosporium* sp. BEL21 isolated from dwarf mistletoe *Arceuthobium oxycedri*. The study of demographic and embryonic parameters of aphids settled on potato plants previously treated with formulations revealed a significant reduction in the numbers of colonizing aphids and a relative increase in the numbers of winged adults, especially in plants treated with BEL21-derived emulsion. The pre-treatment of plants may interfere with and negatively influence embryonic development and early maturity of the embryo and thus affect the fertility of parthenogenetic aphids. BEL21-derived emulsion can ensure effective and an inexpensive control of *M. persicae* for potato spring cropping systems. The current results open real opportunities concerning the implementation of ecofriendly and potent potato protection systems.

## 1. Introduction

Potato (*Solanum tuberoses* L., 1753) production is an agricultural sector of primary importance in Algeria. The government has largely supported its production on a large scale by supporting farmers and contributing to the extension of the planted lands to meet the ever-growing needs of the population. With a production of 4.65 Mt [1], Algeria was ranked as the first potato producer in Africa in 2018. Nevertheless, the development of this crop is often hampered by natural and mainly technical constraints, such as seed availability, weather irregularities, and phytosanitary problems [2].

Late blight caused by *Phytophthora infestans* (Mont.) (de Bary, 1876) and early blight caused by *Alternaria solani* (Sorauer, 1896) is the most important diseases that affect this crop [3]. Aphids (Hemiptera, Aphididae) are also present, but local studies do not provide reliable estimates about the incidence of this insect pest in Algerian potato fields. They can cause significant losses in potato crops by removing plant nutrients by the exhaustion of phloem sap, the stunting of the plant, and leaf deformation, as well as by transmitting several virus diseases [4,5], hindering the development of this strategic sector and forcing massive imports of seed tubers.

The peach potato aphid, *Myzus persicae* (Sulzer, 1776) is polyphagous, feeding on more than 50 plant families, damaging agricultural, industrial, and horticultural crops. It is renowned for its broad host–plant range and resistance to various insecticides [6] and thus is reported on a wide range of crop plants in Algeria [7]. Indeed, *Myzus persicae* affects potato crops, particularly by transmitting viruses (Ex. Potato *Leafroll Polerovirus* and Potato *Virus Y Potyvirus*) [8].

The control of most potato viral diseases is mainly based on the control of aphids as biological vectors, which is possible through the regular application of insecticides. However, the lack of technical management of phytosanitary practices by farmers has led to irrational insecticide use, which often comes with high costs and may lead to many issues, such as secondary pest outbreaks [9,10], the eradication of beneficial fauna [9], environmental contamination, and human health hazards [11,12,13]. To overcome this problem, a reasoned aphid control strategy must be adopted to preserve the agro-ecosystem’s natural equilibrium. The application of ecologically-reassuring biopesticides may be considered as an alternative or integrative to chemical control [14,15]. The effects of endophytic fungi as biocontrol agents against aphids were recently experienced on some crop plants [16,17,18,19,20,21].

The present work aims to test and compare the insecticidal activity against *M. persicae* of culture filtrates derived from three endophytic *Cladosporium* isolates incorporated in invert emulsion formulations. We hypothesize that formulations may negatively affect aphids causing their mortality and reducing their biotic performance but with different degrees concerning the nature and concentration of fungal filtrates. In addition, we suggest that the pre-treatments of the plants by the formulations can negatively affect the biotic potential of the targeted aphid and thus the establishment of its colonies on plants.

## 2. Results

### 2.1. The Aphicidal Activity of Formulations

All formulations have demonstrated insecticidal activity against *M. persicae* individuals. This activity was increased by increasing the fungal filtrate concentration (Table 1). The highest rate of mortality was obtained in treatment with the emulsion containing *Cladosporium* sp. isolate BEL21 filtrate, followed by those of *C. oxysporum* and *C. echinulatum* (for the same concentration). The lowest rate was recorded for the formulation containing *C. echinulatum* filtrate with 44.96% at the concentration of 20%. The results showed that for all formulations the LT50 was negatively correlated with culture filtrate concentration. It is also shown that invert emulsion with BEL21 filtrate has the fastest action (Table 1). Two-way ANOVA and Fisher LSD test demonstrated that the aphicidal activity of the formulations was much more influenced by the concentration (F = 48.79, *p* < 0.0001), as well as by the type of culture filtrate (F = 8.61, *p* < 0.0005). The calculated LC50 differs depending on the nature of the fungal filtrate. Indeed, the least concentrated was recorded for the formulation containing the BEL21 filtrate at 14.53%, followed by that of the inverse emulsion based on *C. oxysporum* filtrate at 22.53%, after which it was slightly more concentrated in the formulation consisting of *C. echinulatum* filtrate, at 24.89%.

### 2.2. Evolution of Chitinolytic Activity of Endophytic Fungi

**Figure 1:**
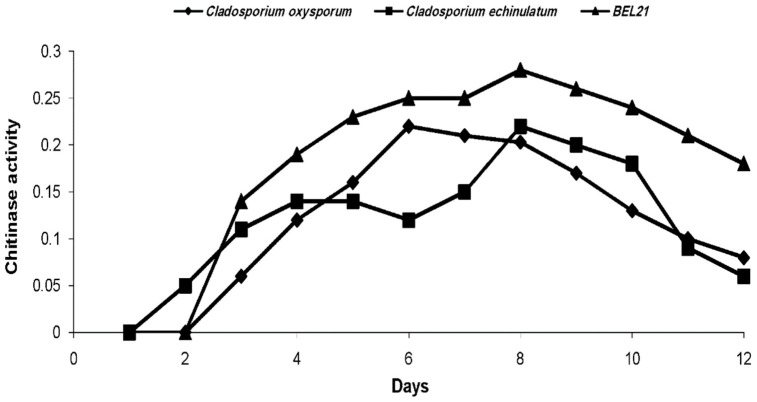
Evolution of chitinase activity in the three endophytic *Cladosporium* (μmol N-acetyl-β-D-glucosamine min·mL^−1^).

The product resulting from the chitinolytic activity of fungi was detected on the second and third days. Product concentration evolved gradually to reach a maximum value on the sixth day for *C. oxysporum* and on the 8th day for *C. echinulatum* and BEL21 filtrates (Figure 1). It has been found that the induction of chitinases is most significant in BEL21 filtrate. Finally, chitinolytic activity declined gradually for all filtrates (Figure 1).

### 2.3. Effects of Plant Pre-Treatment on Aphids’ Demographic and Embryonic Parameters

Regarding the evolution of aphid colonies, it was found that potato plants previously treated with formulations have fewer aphid number compared to untreated plants (Table 2). Mean numbers of first- and third-instar nymphs in plants treated with a formulation containing BEL21 filtrate were, respectively, 3.25 and 2.88 in plants treated by formulation with 20% of culture filtrate and up to 3.13 and 2.13 in plants treated by a formulation containing 80% of culture filtrate. The numbers were lower compared to those recorded in plants treated with formulations containing *C. echinulatum* and *C. oxysporum* filtrates (Table 2).

On the other hand, the number of winged adults was increased in plants treated with BEL21-based formulations (Table 2). Mean numbers of first instar nymphs were more influenced by the fungal origin (F = 14.57, *p* < 0.0001) and fungal filtrate concentration (F = 11.54, *p* < 0.0001), while those of third instar nymphs were more influenced by the concentration of the filtrate (F = 24.86, *p* < 0.0001).

Table 3 demonstrated the effect of potato pre-treatment by invert emulsions on embryonic traits of *M. persicae* apterous adults. The results showed that the embryo number per ovariole decreases in individuals sampled from plants treated with emulsions containing BEL21 filtrate (between 1.83 and 1.51 embryos/ovariole) compared to those sampled from untreated plants (2.37 embryos/ovariole) as well as the number of mature embryos per individual and per ovariole, which declined in dissected aphids sampled from plants treated with emulsions containing BEL21 filtrate (Table 3).

## 3. Discussion

The genus *Cladosporium* was frequently isolated in endophyte form and studied for its various biological virtues [17,22,23]. The natural entomopathogenic activity of the *Cladosporium* taxa was revealed, especially in Hemiptera and Hymenoptera [24,25]. Moreover, some species were tested for insecticidal and entomopathogenic potential against Aleyrodidae [25], Aphididae [17], Tenebrionidae [26], and Bruchidae [27]. The effectiveness was noticed when LT50 is observed to be less prolonged in the BEL21-based emulsion. Bensaci et al. (2015) [17] showed that the mortality of *Aphis fabae* Scop. was positively correlated with the concentration of *C*. *oxysporum* filtrate incorporated in the applied invert emulsion. However, the LT50 was longer, suggesting that the nature of the formulation components, as well as the aphid species, may result in different biological responses. While the fastest action was revealed in the BEL21-based formulation, the least significant LC50 was also found in the latter, indicating a striking aphicidal performance compared to the other formulations. LC50 is a key parameter for evaluating the efficacy of formulations; it was determined by Bensaci et al. (2015) [17] in aqueous suspensions and inverse emulsions based on filtrates and conidial suspensions of *C*. *oxysporum* to evaluate the effectiveness against *A. fabae*. The aphicid activity of formulations was probably attributed to the action of the metabolites contained in the fungal filtrates. Thus, certain toxic compounds, such as cladosporin and cercosporin, are produced by *Cladosporium* [28].

The enzymatic activity is implicated in the nutritional efficiency of fungi. Chitinolytic activity in endophytic *Cladosporium* sp. previously isolated from *N. oleander* was revealed in the framework of the biological control of the bean weevil, *Acanthoscelides obtectus* Say [27]. However, the involvement of this enzymatic activity in aphid mortality has not been proven. Moreover, if the chitinolytic activity was revealed in several endophytic mycotaxa, such as those belonging to the genera *Trichoderma*, *Beauveria*, or *Aspergillus* [29], the link between this activity and the entomopathogenic behaviors of mycoendophytes were advanced by Arnold and Lewis (2006) [30]. Chitinases, produced by *C. oxysporum*, have been demonstrated as a determinative element of fungal virulence against *Toxoptera citricidus* (Kirkaldy) and *T. erytreae* (Del Guercio) [31].

The chitinolytic activity was started between the 1st and 2nd day of incubation for all tested fungi, as previously reported in *C. oxysporum* by Bensaci et al. (2015) [17]. In this comparative context, it is essential to characterize the effectiveness of chitinolytic activity as an entomopathogenic factor concerning targeted aphids through two essential elements: precocity and intensity. Regarding the recorded aphid mortality, the chitinolytic intensity of BEL21 was considerable, but as of late it has been compared to that of *C. echinulatum*. Chitinolytic activity evolved gradually with the incubation time, but this evolution could also be related to other factors, such as pH, temperature, and water activity [29,32].

Potato plants can affect the installation and success of aphids if they find a suitable trophic environment for their development. *M. persicae* cannot be developed or reproduced in the same ways in all host plants, including different potato varieties [33]. We suppose that spraying bio-formulations on plants before their infestations by aphids led to the establishment of an unfavorable phylloplan environment, which can hinder or alter the “test bites” but is not direct repulsion factor. This may explain the negative correlation between the number of individuals per plant and the concentrations of filtrates in sprayed formulations. It is known that winged adults produced in colonies ensure the dissemination flights to colonize new plants. However, the frequency of these aphid morphs depends on abiotic and biotic conditions of the trophic environment [34]. We hypothesize that plant pre-treatment by formulations disrupted the establishment of aphid colonies, consequently speeding up the production of winged adults.

After the dissection of mature *M. persicae* individuals, it was possible to see two ovaries per individual, each of which contained several ovarioles. Their number was stable in all dissected insects, usually falling between 10 and 11 per individual (Table 3). Thus, pre-treatment of potato plants did not affect the ovariole number. In addition, the number of embryos was not affected in aphids developed in plants treated with *C. echinulatum*- and *C. oxysporum*-based formulations. Unlike individuals sampled from plants treated with the emulsion containing BEL21 filtrate, which decreased the number from 27.57 embryos in the untreated plants to 18.00 embryos in specimens sampled from plants treated by formulations with the highest filtrate concentration (80%). However, for aphids sampled from the other plants, pre-treatments did not result in a decrease in embryo number. On the other hand, we found that embryo number per ovariole decreases in individuals sampled from plants treated with emulsions containing BEL21 filtrate (between 1.83 and 1.51 embryos/ovariole) compared to those sampled from untreated plants (2.37 embryos/ovariole) as well as the number of mature embryos per individual and per ovariole, which declined in dissected aphids sampled from plants treated with emulsions containing BEL21 filtrate (Table 3).

The mean number of *M. Persicae* ovarioles settled on the pre-treated plants was stable. The number of ovarioles in *Acyrthosiphon pisum* (Harris) was generally between 6 to 10 [35], whereas Takada (1984) [36] found that this number in *M. persicae* grown on radish ranged from 10 to 18. Ovariole number is an important index that determines the reproductive success of the aphid, but it is linked to intrinsic factors, such as photoperiodism and the nutritional quality of the host plant [37]. We consider that ovariole ontogeny is not affected by the applied formulations.

The negative influence of the pre-treatment was noticed for all formulations on the number of mature embryos, especially for the individuals developed on the plants treated with formulations based on BEL21 filtrate, so we can deduce that the preliminary treatments of plants led to an alteration of the embryonic development of aphids, probably by a slowing of the embryogenesis process. This phenomenon probably limits the fertility of aphids.

## 4. Material and Methods

### 4.1. Plant Material

Potato plants (cv. Arinda) were used for all experiments. They were planted in pots (35 cm × 25 cm) containing the base gravel superimposed by a growing substrate composed of previously dry-sieved (2 mm) soil and sand (2:1), which were autoclaved for 40 min at 121 °C and 15 p.s.i. Plants were kept in an experimental greenhouse (210 m^2^, 28 °C) for 32 days preceding the beginning of the experiments, and covered with plastic cylinders lined with muslin to prevent the entry of other aphids or their natural enemies. Tubers were obtained from the National Center for Control and Certification of Seeds and Plants (CNCC).

### 4.2. Aphids

Individuals of M. persicae were obtained from a colony maintained on potato plants, cv. “Arinda” was established from a single parthenogenetic apterous female collected from *Malva sylvestris* L., 1753. Nymphs were repeatedly transferred to the same potato genotype to obtain an adapted aphid strain for each host plant.

### 4.3. Isolation and Preservation of Endophytic Fungi

Three fungal endophytes belonging to the genus *Cladosporium* (Link, 1816) were used in the present study: *Cladosporium echinulatum* (Berk.) (G.A. de Vries, 1952) and *C. oxysporum* (Berk. and M.A. Curtis, 1868), were isolated respectively from healthy leaves of *Nerium oleander* (L., 1753) and *Euphorbia bupleuroides* subsp. *luteola* (Kralik) (Maire, 1939), which had been identified based on their morphological traits [38,39]. The third *Cladosporium*, which has not been identified at the species level, was isolated from shoots of the dwarf mistletoe, *Arceuthobium oxycedri* (DC.) (M. Bieb., 1819). The identification of this mycotaxon was achieved by molecular characterization.

DNA extraction was performed according to Lee and Taylor (1990) [40] using as lysis buffer: Tris-HCl (50 mM); EDTA (50 mM); 3% SDS; and 1% 2-mercaptoethanol, TE-saturated phenol, NaOAc (3 M)/pH: 8.0, and Isopropanol and Ethanol (70%), while the PCR amplification of fungal DNA was performed according to White et al. (1990) [41]: targeting ITS1 5.8S (5′-TCCGTAGGTGAACCTGCGG-3′) and ITS2 (5′-GCTGCGTTCTTCATCGATGC-3′) genomic units (O’Donnell et al. 2000) using ProFlexTM PCR System (Applied Biosystems); in addition to matrix (extraction) DNA (1 μL), the reaction mixture was composed of an amplification buffer (10× buffer (Sigma-Aldrich, Burlington, MA, USA)—100 mM Tris-HCl, pH 8.3 at 25 °C, 500 mM KCl, 15 mM MgCl_2_, 0.01% gelatin), dNTP CleanAMP (10 mM for each dNTP) (Sigma-Aldrich), DNA Polymerase from *Taq* SuperPak (Sigma-Aldrich) compatible with MgCl_2_ buffer, ultra-pure sterile water, and primers. After testing the PCR product in 1% agarose gel (with Tris-borate-EDTA as a buffer) and DNA purification using QiaQuick Gel Extraction kit (Qiagen, Germany), the targeted units have been sequenced in ABI-Prism 373A DNA sequencer (Applied Biosystems). Obtained sequences were aligned using a Bioedit Sequence Alignment Editor 7.0.0. [42], deposited at GenBank and recorded as accession number MH760413, authenticated as *Cladosporium* sp. isolate BEL21.

Fungal isolates were stored in tubes containing silica gel [43] and then transferred in Petri dishes containing Malt Extract Agar medium supplied with tetracycline (50 µg·mL^−1^).

### 4.4. Culture Filtrates Preparation

The peripheral fragments of Cladosporium colonies were separated and transferred to Wickerham liquid medium [44]. They were placed in 500 mL Erlenmeyer flasks and incubated in the dark at 24 °C. After 3 days and once a week, 1 g of glucose was added [45,46]. The Erlenmeyer flasks underwent regular agitations (150 tpm) for two hours and twice a day to homogenize their content. After 17 days, fungal suspensions were recovered, centrifuged at 6000 rpm for 15 min, and aseptically filtered through no. 1 and no. 2 Whatman paper on a borosilicate glass funnel. The filtrates were kept at a low temperature (2 °C) for later use [17].

### 4.5. Formulations Conception

An inverted emulsion was prepared for each fungal filtrate. Before incorporating them into the formulation, fungal filtrates were first placed in a glass dish (45 × 25 × 30 cm^3^) under a temperature set at 15 °C for 12 h. The formulation was composed of two phases: the aqueous phase containing fungal culture filtrate amended with salicylic acid (0.5 g/500 mL water) and 0.01% glycerol, and the oily phase that comprises a vegetable oil with low viscosity to which Tween 80 [17] and 1.1% sodium alginate (*w*/*v*) was added. Both phases were homogenized mechanically using a mechanical stirrer with a propeller to obtain a pale yellow emulsion. Each formulation was prepared according to a concentration gradient of fungal filtrates (20, 40, 60, and 80%) then placed in a 500 mL fumed glass flask to avoid photochemical alteration, and then they were preserved in a refrigerator (4 °C) before their subsequent use.

### 4.6. The Aphicidal Activity of Formulations

For the experimental treatments of aphids, the ventilated chamber bioassay model (VCB) was adopted [47]. The targeted aphids were placed in detached potato leaflets, sheathed at petiolules by cotton soaked in a modified mineral solution of McArthur and Knowles (1992) [48] (20 mM Ca(NO3)_2_; 30 mM KNO_3_; 20 mM MgSO_4_; 185 μM H_3_BO_3_; 36.5 μM MnCl_3_; 0.3 μM ZnSO_4_; and 0.3 μM FeSO_4_·7H_2_O). Leaflets were enclosed in perforated glass boxes. For each concentration, six glass boxes were used, each of which contained 20 aphids.

Aphids were sprayed with 5 mL of formulated emulsion in each treatment in the late morning using a manual pressure sprayer under a laboratory temperature of 27 °C. Insect spraying was performed once only, thereafter the boxes were closed. Six other glass boxes were left with nontreated individuals as a control experiment. Within 10 h after treatments, aphid mortality was recorded after each hour. Corrected mortality was calculated according to the formula of Abbott (1925) [49] given as follows:

Mortality (%) = (X − Y/100 − Y) × 100,(1)

where X is the percentage of mortality in the treated samples and Y represents the average percentage mortality of the control unit.

Lethal concentration 50 (LC50) and lethal time 50 (LT50) were calculated after the transformation of mortality data into probit, according to Finney (1971) [50].

### 4.7. Evolution of Chitinolytic Activity of Endophytic Fungi

The calculation of colony disks from Petri dishes were taken from the edges and deposited in flasks containing a culture medium with colloidal chitin (Sigma-Aldrich) as a substrate containing 1 L: 4 g colloidal chitin; 0.7 g K_2_HPO_4_; 0.5 g MgSO_4_·5H_2_O; 0.3 g KH_2_PO_4_; 0.01 g FeSO_4_·7H_2_O; 0.5 g peptone; 1 mg MnCl_2_; and 1 mg ZnSO_4_ [17,21]. The medium was dark-incubated at 24 °C with regular stirring for 30 min (150 rpm) every 2 h [17]. After cold centrifugation (6000 rpm at 4 °C for 30 min) [51] the supernatant was filtered through no. 2 Whatman paper. Every 24 h, a volume of 0.9 mL of colloidal chitin mixed with sodium acetate solution (50 mM) was added to 0.1 mL of the filtered supernatant. After one hour of incubation at 37 °C 0.2 mL of NaOH (1N) was added to the mixture, after which centrifugation (8000 rpm for 8 min) was performed and the supernatant was recovered for the determination of N-acetyl-β-D-glucosamine using a Ultraviolet/visible spectrophotometer [52]. The reaction product (Nacetyl-β-D-glucosamine) was determined every 24 h for 12 days.

### 4.8. Effects of Plant Pre-Treatment on Aphid’s Demographic and Embryonic Parameters

In another experiment, potted potato plants with 4 to 6 leaves were sprayed with the same formulations (inverted emulsions containing *C. echinulatum, C. oxysporum*, and *Cladosporium* BEL21 filtrates). After 2 h, all stems were eliminated except one, on which three *M. persicae* nymphs were deposited at the apex. The infected plants were covered with plastic cylinders lined with muslin. Aphid colonies were monitored for 18 days to evaluate the state of demographic development. First and third nymph instars and the total number of apterous and winged adults are counted on each plant. Eight potato plants were tested per treatment for each filtrate concentration, while nine untreated plants (three per treatment lot) were infested using the same method as the previous ones and held as a control.

To study the effect of plant pre-treatment on embryo production in parthenogenetic females, apterous adults have been dissected in a 70% methanol [53] and then observed using a light microscope. Mean embryo and ovarioles number per adult, mean embryo number per ovariole, mean mature embryo (with pigmented eyes) per adult aphid, and per ovariole were recorded.

### 4.9. Statistical Analysis

The effects of direct treatment on aphid mortality and the pre-treatment of potato plants on aphids’ demographic and embryonic parameters were compared by performing a separate two-way ANOVAs followed by the Fisher LSD test when significant treatment effects were found (*p* ≤ 0.05). LC50 and LT50 were calculated using probit–logit analysis performed by XLSTAT Pro 2014.5.03 (©Microsoft Office).

## 5. Conclusions

The findings suggested that inverted emulsions based on culture filtrates from endophytic *Cladosporium* are considered effective formulations to control *M. persicae* on potato plants. In addition, the early application of these bioproducts on host plants can disrupt the biotic and demographic performance of this pest. In this respect, it is not only recommended to improve these formulations but also to multiply the treatment modalities as was performed on the seeds or, alternatively, to test the volatile action in order to determine their repulsive effects against aphids.

## Figures and Tables

**Table 1:** Aphicid activity and LT50 of the designed bioformulations against *M. persicae*.

Filtrate Concentration (%)	*Cladosporium echinulatum*		*Cladosporium oxysporum*		*Cladosporium* BEL 21	
	CM (%) 1	LT50 (h)	CM (%) 1	LT50 (h)	CM (%) 1	LT50 (h)
20	44.96 ± 5.63 ^c^	15.71	45.79 ± 5.58 ^c^	16.64	61.63 ± 5.26 ^c^	10.03
40	53.29 ± 4.94 ^bc^	15.65	64.13 ± 6.72 ^b^	9.52	74.96 ± 4.90 ^bc^	6.93
60	72.46 ± 6.04 ^ab^	7.11	79.96 ± 4.74 ^ab^	7.01	84.96 ± 4.94 ^ab^	5.15
80	92.46 ± 22.14 ^a^	4.14	93.29 ± 3.01 ^a^	4.44	94.96 ± 2.48 ^a^	2.78
Control	3.34 ± 0.72	/	2.50 ± 0.88	/	1.67 ± 0.34	/

1 Corrected mortality. Values are means ± SE. Means followed by the same letter(s) within the same column are not significantly different (α = 0.05) according to LSD Fisher test.

**Table 2:** Effect of potato pre-treatment by invert emulsions on demographic parameters of *M. persicae*.

**Filtrate Concentration (%)**	***Cladosporium echinulatum***			
	**1st Instar Number**	**3rd Instar Number**	**Apterous Adult Number**	**Winged Adult Number**
20	5.37 ± 1.86 ^bc^	6.38 ± 0.85 ^bc^	3.25 ± 0.66 ^b^	0.25 ± 0.43 ^bc^
40	5.12 ± 2.14 ^bc^	5.00 ± 1.41 ^de^	2.75 ± 1.47 ^bc^	0.25 ± 0.43 ^bc^
60	5.25 ± 2.10 ^bc^	4.87 ± 1.16 ^de^	2.88 ± 0.92 ^bc^	0.37 ± 0.48 ^bc^
80	4.50 ± 1.80 ^cd^	4.62 ± 1.49 ^de^	3.38 ± 0.99 ^b^	0.62 ± 0.48 ^ab^
Control	9.00 ± 1.63 ^a^	8.00 ± 3.74 ^b^	6.33 ± 1.69 ^a^	0.00 ^c^
**Filtrate Concentration (%)**	***Cladosporium oxysporum***			
	**1st Instar Number**	**3rd instar Number**	**Apterous Adult Number**	**Winged Adult Number**
20	5.63 ± 0.85 ^bc^	3.88 ± 0.7 ^efg^	2.63 ± 0.69 ^bc^	0.13 ± 0.33 ^c^
40	4.88 ± 1.26 ^bcd^	5.25 ± 1.56 ^cd^	2.88 ± 0.78 ^bc^	0.13 ± 0.33 ^c^
60	5.38 ± 1.72 ^bc^	4.25 ± 1.29 ^def^	2.75 ± 0.66 ^bc^	0.25 ± 0.43 ^bc^
80	5.75 ± 1.85 ^bc^	4.63 ± 0.99 ^de^	3.50 ± 0.86 ^b^	0.25 ± 0.43 ^bc^
Control	7.00 ± 0.81 ^ab^	7.33 ± 1.24 ^b^	5.00 ± 0.81 ^a^	0.00 ^c^
**Filtrate Concentration (%)**	***Cladosporium* BEL21**			
	**1st Instar Number**	**3rd Instar Number**	**Apterous Adult Number**	**Winged Adult Number**
20	3.25 ± 0.66 ^ef^	2.88 ± 0.59 ^gh^	2.88 ± 0.78 ^bc^	0.25 ± 0.43 ^bc^
40	2.75 ± 0.82 ^f^	3.12 ± 0.92 ^fgh^	3.13 ± 0.92 ^bc^	0.62 ± 0.48 ^ab^
60	3.50 ± 0.86 ^def^	2.25 ± 0.43 ^h^	2.75 ± 0.82 ^bc^	0.50 ± 0.50 ^abc^
80	3.13 ± 1.05 ^ef^	2.12 ± 0.59 ^h^	2.13 ± 0.92 ^c^	0.87 ± 0.59 ^a^
Control	9.00 ± 1.63 ^a^	11.00 ± 2.16 ^a^	2.67 ± 0.94 ^bc^	0.00 ^c^

Values are means ± SE. Means followed by the same letter(s) within the same column are not significantly different (α = 0.05) according to LSD Fisher test.

**Table 3:** Effect of potato pre-treatment by invert emulsions on embryonic traits of *M. persicae* apterous adults.

**Filtrate Concentration (%)**	***C. echinulatum***				
	**Ovariole Number**	**Embryo Number**	**Embryo Number/Ovariole**	**Mature Embryo Number**	**Mature Embryo Number/Ovariole**
20	11.43 ± 1.17 ^ab^	26.43 ± 4.95 ^ab^	2.30 ± 0.24 ^abc^	5.71 ± 1.57 ^bcde^	0.51 ± 0.13 ^bcde^
40	11.71 ± 0.88 ^ab^	29.57 ± 1.76 ^a^	2.54 ± 0.21 ^a^	6.71 ± 1.48 ^abcd^	0.58 ± 0.15 ^abcd^
60	11.29 ± 0.88 ^ab^	21.43 ± 3.73 ^def^	1.90 ± 0.31 ^def^	7.14 ± 1.24 ^ab^	0.64 ± 0.13 ^abc^
80	11.71 ± 0.69 ^ab^	27.29 ± 5.00 ^ab^	2.35 ± 0.33 ^abc^	6.71 ± 1.27 ^abcd^	0.58 ± 0.11 ^abcd^
Control	11.43 ± 0.90 ^ab^	24.14 ± 2.23 ^bcd^	2.12 ± 0.19 ^cde^	7.71 ± 1.66 ^a^	0.66 ± 0.10 ^a^
**Filtrate Concentration (%)**	***C. oxysporum***				
	**Ovariole Number**	**Embryo Number**	**Embryo Number/Ovariole**	**Mature Embryo Number**	**Mature Embryo Number/Ovariole**
20	10.71 ± 0.88 ^b^	26.29 ± 2.71 ^ab^	2.45 ± 0.14 ^ab^	7.57 ± 0.72 ^a^	0.71 ± 0.07 ^a^
40	11.43 ± 0.49 ^ab^	26.57 ± 2.71 ^ab^	2.38 ± 0.23 ^abc^	6.86 ± 0.83 ^abc^	0.60 ± 0.08 ^abc^
60	11.71 ± 1.03 ^ab^	26.29 ± 3.14 ^ab^	2.24 ± 0.14 ^bc^	5.29 ± 1.57 ^de^	0.46 ± 0.12 ^de^
80	11.14 ± 0.98 ^ab^	23.71 ± 1.97 ^bcde^	2.14 ± 0.20 ^cd^	4.71 ± 0.69 ^e^	0.43 ± 0.08 ^e^
Control	10.71 ± 0.88 ^b^	25.43 ± 4.40 ^bc^	2.38 ± 0.40 ^abc^	6.86 ± 1.88 ^abc^	0.65 ± 0.18 ^ab^
**Filtrate Concentration (%)**	***Cladosporium* BEL21**				
	**Ovariole Number**	**Embryo Number**	**Embryo Number/Ovariole**	**Mature Embryo Number**	**Mature Embryo Number/Ovariole**
20	11.29 ± 0.69 ^ab^	20.71 ± 3.69 ^def^	1.83 ± 0.28 ^f^	5.57 ± 0.90 ^cde^	0.50 ± 0.09 ^cde^
40	11.57 ± 0.49 ^ab^	21.86 ± 2.23 ^def^	1.84 ± 0.16 ^ef^	5.29 ± 1.48 ^de^	0.45 ± 0.11 ^de^
60	11.43 ± 1.04 ^ab^	20.14 ± 3.18 ^ef^	1.76 ± 0.18 ^fg^	4.71 ± 1.27 ^e^	0.42 ± 0.13 ^e^
80	12.00 ± 0.75 ^a^	18.00 ± 2.82 ^f^	1.51 ± 0.27 ^g^	4.43 ± 0.90 ^e^	0.37 ± 0.09 ^e^
Control	11.43 ± 1.04 ^ab^	27.57 ± 4.30 ^ab^	2.37 ± 0.19 ^abc^	6.71 ± 1.66 ^abcd^	0.58 ± 0.15 ^abcd^
*p*	0.504	<0.0001	<0.0001	<0.0001	<0.0001

Values represent means ± SE. Means followed by the same letter(s) within the same column are not significantly different (α = 0.05) according to LSD Fisher test.

## Data Availability

Not applicable.
